# A Survey of Neonatal Nurses Perspectives on Voice Use and Auditory Needs with Premature Infants in the NICU

**DOI:** 10.3390/ijerph18168471

**Published:** 2021-08-11

**Authors:** Amy R. Smith, Deanna Hanson-Abromeit, Ashley Heaton, Brenda Salley

**Affiliations:** 1 Department of Music Therapy, School of Music, Sam Houston State University, Huntsville, TX 77340, USA; 2 Children’s Mercy Research Institute, Kansas City, MO 64108, USA; 3 Department of Music Education and Music Therapy, School of Music, University of Kansas, Lawrence, KS 66045, USA; dhansonabromeit@ku.edu; 4 Children’s Memorial Hermann Hospital, Houston, TX 77030, USA; ashley.heaton@memorialhermann.org; 5 Department of Pediatrics, University of Kansas Medical Center, Kansas City, KS 66160, USA; bsalley@kumc.edu; 6 Department of Pediatrics, Children’s Mercy Hospital, Kansas City, MO 64108, USA

**Keywords:** prematurity, auditory development, language development, voice use, language environment, auditory environment, nursing

## Abstract

Background: Exposure to the voice and language during the critical period of auditory development associated with the third trimester is thought to be an essential building block for language. Differences in the auditory experience associated with early life in the NICU may increase the risk of language delays for premature infants. NICU nurses are fundamental in the care of premature infants; how they use their voices may be important in understanding auditory experiences in the NICU. This study examined voice use behaviors of NICU nurses in the United States and their current knowledge of early auditory development. Method: An opt-in, online questionnaire. Results: Nurses reported using their voice more as the age of infants approached term gestation and speaking to infants was the most common type of voice use. Both infant and nurse factors influenced reported voice use decisions in the NICU. Nurses did not believe the NICU auditory environment to be sufficient to meet early auditory needs of premature infants but did believe that premature infants are exposed to adequate voice sounds. Conclusions: A gap in knowledge regarding the importance of early exposure to voice sounds may be a barrier to nurses using their voices to support early auditory development.

## 1. Introduction

Language development is an intricate process that requires an organized, consistent relationship between early auditory experiences, exposure to language, and opportunities for reciprocal communication [1,2]. Auditory perceptual development begins during the third trimester and continues into early infancy, building the foundation for language development [3,4]. Hearing language in utero builds the infant’s capacity to recognize patterns and sounds that are characteristic within the primary language [5]. Language development is highly dependent upon stimulation, first from the intrauterine environment, and, in turn, exposure to an environment rich in vocabulary, infant-directed speech, and social exchanges [6,7,8,9,10,11]. Infants born premature (i.e., before 37–38 weeks gestational age) and hospitalized in the Neonatal Intensive Care Unit (NICU) miss out on important intrauterine auditory experiences [12]. Additionally, environmental and medical needs associated with premature birth may be a barrier to the early language environment provided by close contact with parents that is afforded in healthy term infants. Caregivers, such as nurses, may play an important role in providing early exposure to language by using their voice while providing care to premature infants in the NICU. Early language-rich interactions between infants and caregivers support critical brain development that is necessary for optimal language and long-term economic and health outcomes [10]. These differences associated with early life in the NICU may put premature infants at a disadvantage for altered auditory perceptual development and elevate their risk for less optimal language outcomes [2,13]. Understanding infant and environmental factors that influence nurses’ decisions to use their voice (and thus provide auditory and language input to the infant) is a foundational step for efforts aimed at optimizing the auditory environment of the NICU.

A substantial amount of research suggests that the auditory development of premature infants is directly impacted by the NICU environment [12,13,14,15,16]. Altered cerebral development, a possible result of the stressful environment of the NICU, has been linked to slower processing of auditory input, which may impact language outcomes in premature infants [13,17,18]. In contrast to the NICU, the intrauterine soundscape is rich with frequencies and prosodic characteristics of language that is carried by both the mother’s voice and other voices in the environment. Intrauterine exposure to the voice and language characteristics are thought to be essential for the onset of neural organization of the auditory system during the third trimester and create a potent pathway for rapid processing of voice sounds [6,19,20]. Notably, it is still unknown how missing such intrauterine auditory experiences may or may not compromise premature infants’ auditory development and language development capacity. Importantly, we also do not know what types of auditory experiences (e.g., optimal amount of speech, music, or other sounds) are necessary to best support early auditory and language development in the NICU—a question that is further complicated by the complexities of the infants’ medical stability, gestational age, and competing NICU auditory sensory experiences.

Infants who are born premature may experience very different caregiving interactions in the NICU compared to infants who are born full term due to their medical fragility and underdeveloped sensory system. They are frequently protected from the kinds of auditory input they may have received in utero or that is typical for full-term infants. Within the NICU there is a focus on reducing sounds known to be harsh or negative to the still developing sensory systems of premature infants. It remains an uncommon practice to add sounds, even those that may be beneficial to auditory development, into the NICU environment. In comparison to full-term infants whose postnatal environment allows for routine caregiver interactions, premature infants often have less access to parent voices and infant-directed interactions due to medically necessary care [21]. Very premature infants may be in a type of protective bed (i.e., an isolette) that presents a physical barrier, making interaction and voice exposure more difficult. Recognizing these barriers is critical, because exposure to infant-directed speech (with its characteristically exaggerated prosody and ability to engage infant attention) and responsive interactions (contingent responses to infants’ behavioral cues such as eye contact, facial expression, and vocalizations) are essential for optimizing early language and development [22].

Maximizing parent involvement in the NICU is widely accepted as being important for developmental outcomes of premature infants. Encouraging family caregivers to use their voices (speaking, reading, and singing) with their premature infants is commonly accepted and valued in the NICU because of the known positive impact it can have on overall health outcomes and long-term developmental milestones such as language [23]. Caregivers speaking, reading, and singing to infants in the NICU has not been associated with higher ambient sound levels, making it a safe addition to infant care [24,25].

Interventions in the NICU that specifically involve increasing exposure to language-related experiences have primarily focused on the mother’s voice. Particularly in the United States, socio-economic resources, as well as social (e.g., maternal age, marital status, insurance, and childcare for other children) and psychological factors are barriers that may prevent consistent contact between infants in the NICU and family caregivers [21,26]. Less attention has been given to the role of health providers in the NICU as potentially valuable sources for providing auditory/language input experiences to infants.

As key stakeholders in the NICU, nurses are responsible for all aspects of bedside care, including the management of environmental characteristics such as sound and interaction. Nurses make ongoing decisions about their own interactions as well as guide the timing and acceptability of other professional interventions. Neonatal nurses continuously contribute to the premature infant caregiving environment in the NICU as they interact with premature infants while performing a variety of medical tasks. They are trained to consider multiple characteristics of the infant and environment when making decisions about their own interactions, including the manner in which they use their voices. The frequency and duration with which nurses are in direct contact with premature infants may make their role in early language development more important than previously considered. Prior to developing and evaluating effective interventions for auditory/language experiences for premature infants in the NICU, it is critical to first identify neonatal nurses’ practice and perceptions of voice use in the NICU. How nurses use their voice based on environmental and infant factors, what nurses know about early auditory development, and individual nurse factors, such as experience and age, are all foundational components of inquiry important to shaping understanding of this topic. To our knowledge, there have been no previous studies that have examined nurses’ use of their own voices when caring for premature infants, or that have examined what nurses believe about the auditory development/needs of premature infants and how their voices may contribute to auditory/language experiences.

The current foundational study aims to address this gap by identifying and describing the beliefs that neonatal nurses have about auditory needs of premature infants and understand how nurses use their voice when interacting with premature infants. We define *voice use* as a person’s use of their voice for an auditory output, including speaking (to an infant; to another adult), singing, humming, or whispering in real time. To investigate nurses’ beliefs about voice use and auditory development, as well as their voice use behaviors in the NICU, we conducted a survey of NICU nurses to examine the following research questions: (1) How do nurses use their voice when interacting with premature infants? (2) Do infant characteristics (age, medical stability, behavior state) influence the voice behaviors of NICU nurses? (3) What do NICU nurses perceive to be the auditory needs of premature infants? (4) Do NICU nurse characteristics (age and years of experience) influence their beliefs or voice behaviors?

## 2. Materials and Methods

Our target population was nurses currently working in the NICU in the United States (U.S.). Nurses who met the following criteria were eligible to participate in this survey study: (1) currently employed full-time or part-time in a neonatal intensive care unit in the U.S.; and (2) hold an active license as a registered nurse (RN). The Institutional Review Board at Sam Houston State University (Houston, TX) approved this study on 3 July 2020.

To our knowledge, there are no previous surveys that have been used to investigate the voice use behaviors or auditory development beliefs of nurses in the NICU. Questionnaire items were constructed by the study team based on professional experience and content expertise. The questionnaire was organized into the following topic areas: (1) demographics and professional background; (2) characteristics of the NICU; (3) voice use in the NICU; (4) beliefs about auditory development; and (5) experiences with music and exposure to music therapy. Prior to its use in the current study, five NICU nurses tested the questionnaire for clarity, ease of use, and estimated completion time (10–15 min) using the online delivery platform. The development process resulted in a 55-item online questionnaire hosted by an online survey platform (Qualtrics©, Provo, UT, USA). The questionnaire included ordinal, nominal, and open-ended responses. Participants were able to skip questions and some questions allowed multiple responses.

A convenience sample of neonatal nurses was collected through the use of social media, personal networks of the research team, and snowballing as strategies to survey potential participants using the opt-in online questionnaire. The questionnaire was distributed two times. The first questionnaire was distributed to nurses in the personal network of the researchers through email and Facebook posts. The online link could be shared, and participants were encouraged to send it to others in their own personal network. The first distribution lasted two weeks with a reminder email and Facebook post sent at the beginning of the second week. A preliminary review of the results from the first distribution led the research team to make minor revisions to the questionnaire to improve clarity before additional participants were recruited. Specifically, two questions were changed from a sliding scale to a Likert response option for more consistency across responses. Two additional questions were added to the revised questionnaire to understand the role of parents related to infant auditory needs and to understand nurses’ voice interactions with infants compared to adults. See Supplementary Materials for the specific revisions and the revised questionnaire used on the second distribution. The revised questionnaire was then distributed to three Facebook groups designed specifically for NICU nurses: (1) Neonatal ICU Nurses Rock, (2) NICU Professionals, and (3) NICU Nurses. The survey link was posted on each group page. The survey remained open for two weeks and reminder messages were posted to each Facebook page at the start of the second week.

Participants who selected the link in both distributions were directed to an online informed consent page that explained the purpose of the study and contained the contact information for the PI and IRB. Next, participants answered questions to confirm their eligibility. When participants finished the questionnaire, they were directed to a confirmation/thank you page prior to exiting the online platform.

## 3. Results

There was a combined total of 82 responses collected between the first and second distribution of the survey. Seven responses were excluded due to the participants working outside the U.S. The remaining 75 responses were included in analyses. A response rate was not available due to use of open link and social media recruitment. Results from the first sample (*n* = 37) were compared to the second sample (*n* = 38). Both produced nearly identical distributions of answers across all questions, which is an indicator that survey responses are representative of the population of interest. Comparing two survey samples is an alternative approach to determining the representativeness of a survey when response rates are low or cannot be calculated [27].

NICU nurse respondents in the current study were demographically similar to the workforce of NICU nurses in the U.S in terms of age, race, gender, and education [28]. A full summary of participant demographics is given in Table 1. Unit characteristics of the sample included representation from all NICU designs and levels (details regarding unit characteristics are in Table 2). Responses were collected across all regions of the United States.

### 3.1. Voice Use Behaviors of NICU Nurses

Nurses were asked about all the ways they *typically* use their voice while caring for premature infants in the NICU. During typical daily care, the majority of nurses reported talking directly to infants during their routine interactions (*n =* 42, 82.4%). About half of the respondents indicated whispering to infants (*n* = 27, 52.9%), and a smaller proportion of nurses reported singing (*n =* 18, 35.3%) or humming (*n* = 13, 25.5%) to infants as part of their routine care. A subset of nurses indicated that they typically stay silent/do not use their voice with the infants (*n* = 14, 27.5%). The majority of nurses reported that during typical care with infants they also talk to other adults (*n* = 36, 70.6%), while a smaller proportion reported whispering to other adults (*n* = 15, 29.4%).

Nurse respondents were asked about which situations/daily tasks in the NICU they typically use their voice. The questionnaire allowed the participants to select multiple situations/daily tasks. A majority of nurses reported using their voice during diaper changes (*n* = 45, 93.8%), feeding (*n* = 42, 87.5%), and medical procedures (*n* = 38, 79.2%). Nurses reported being less likely to use their voice while taking vital signs (*n* = 22, 45.8%) or giving medication (*n* = 19, 39.6%). Only one respondent indicated they did not use their voice at all during infant interactions. Almost all nurses reported changing the sound of their voice in some way when interacting directly with a premature infant compared to when they are talking to an adult (*n* = 25, 96.2%), although this question was only included in the second version of the questionnaire.

### 3.2. Influence of Infant Characteristics on Nurse Voice Use

Nurse respondents were asked about which infant characteristics typically influence their voice use behaviors during routine care in the NICU. Almost all nurses reported their voice use was always influenced by medical stability (*n* = 53; 93%), behavior cues, such as restlessness, crying, or alertness (*n* = 50, 89.6%), and infant age (*n* = 49; 86%). Nurse respondents reported they are more likely to use their voice as the medical stability of the infant improves. Respondents reported they were unlikely (*n* = 34, 61.8%) to use their voice with an infant that is medically unstable, but likely to use their voice with infants who are variably stable (*n* = 45, 83.3%) or stable (*n* = 55, 100%).

**Figure 1:**
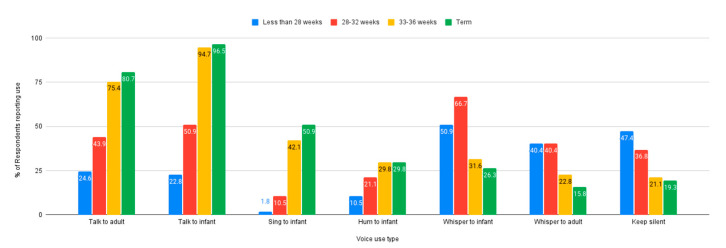
Type of voice use reported by infant gestational age.

Nurses were asked about the influence of gestational age of the infant on their voice use in the NICU; how nurses use their voice seems to be impacted by gestational age. A higher incidence of whispering to the infant and keeping silent was reported for infants who are less than 28 weeks gestational age, while talking to the infant or another adult was reported as much lower in the same age range. In comparison, almost all nurses reported talking to either the infant or another adult when caring for infants who are term. Figure 1 illustrates how types of voice use were reported according to gestational age.

### 3.3. Nurse Knowledge and Beliefs about Premature Infant Auditory Development

Respondents were asked about the role of environmental characteristics on premature infants’ auditory development. Half of nurses (*n* = 25, 50%) reported they do not believe the overall NICU environment is sufficient to meet the auditory needs of premature infants. However, when asked specifically about voice sounds in the environment, just over half of respondents agreed (*n* = 26, 52%) that premature infants have sufficient exposure to voice sounds. A majority of nurse respondents indicated they thought background noise prevented infants from hearing voice sounds (*n* = 36, 84%) and over half of respondents indicated that silence does not prevent infants from sufficient exposure to voice sounds (*n* = 29; 58%). A higher proportion of nurses indicated that they believed parents provide sufficient voice exposure for their infant (*n* = 21, 42%), compared to those who disagreed (*n =* 16, 32%). The majority of nurses reported that parents were frequently present in the NICU most days of the week and/or for a long duration of time (*n* = 16, 60%; in revised questionnaire), whereas 40% (*n* = 10) reported parents were present a few days a week and/or for variable duration of time.

We examined the influence of unit design on beliefs about premature auditory needs. Survey respondents were closely distributed across three different unit types: open bay (*n* = 18, 28.6%), private room (*n* = 23, 36.5%), and combination (*n* = 22, 34.9%). The majority of respondents from both open bay (*n* = 7, 58.3%) and combination (*n* = 10, 55.6%) units did not believe the overall environment was sufficient to meet infants’ auditory needs. In contrast, among nurses in a private room NICU, half believed the auditory environment was sufficient (*n* = 8, 40%) and half believed it to be insufficient (*n* = 8, 40%). Regardless of unit type, participants endorsed the belief that premature infants are exposed to a sufficient amount of voice sounds in the NICU. Similarly, unit type did not influence the belief that background noise prevents infants from hearing voice sounds, and that silence does not prevent sufficient exposure to voice sounds. However, nurse perceptions of whether or not parents provide sufficient exposure to voice sounds did vary by unit design. Half of respondents from open bay units did not believe parents provide sufficient exposure to voice sounds (*n* = 6, 50%) while the majority of respondents from private rooms (*n* = 12, 60%) believed parents do provide sufficient voice exposure. Respondents from combination units reported a more equal distribution between agree (*n* = 4, 25.1%), neutral (*n* = 6, 37.5%), and disagree (*n* = 6, 37.5%) regarding parents providing sufficient voice exposure.

**Figure 2:**
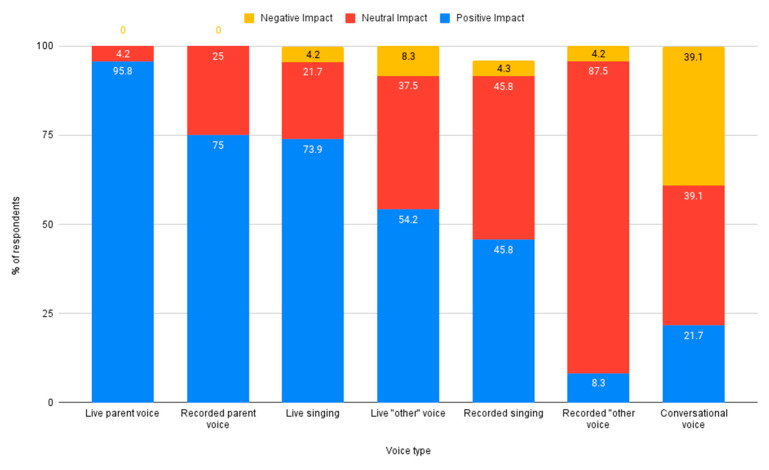
Reported impact of voice types on auditory development.

**Figure 3:**
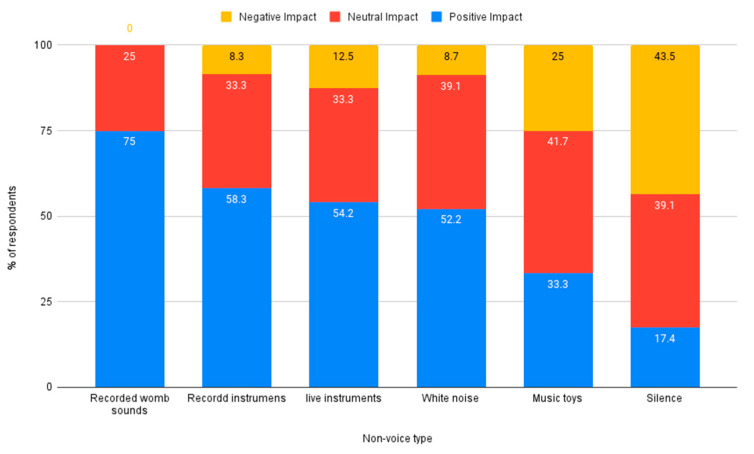
Reported impact of non-voice sounds on auditory development.

Nurses were asked to rate the impact (positive, neutral, or negative) of different sound types on the auditory development of premature infants. Voice sounds such as speaking and singing were rated higher overall, compared to non-voice sounds such as womb sounds or instrumental music. Voice sounds rated by respondents as having the most positive impact on auditory development were live parent voice, recorded parent voice, and live singing. The voice sound rated as having the most negative impact on auditory development was conversation, see Figure 2. Non-voice sounds rated by respondents as having the most positive impact were recorded womb sounds and recorded instrumental music. The non-voice sound rated as having the most negative impact on auditory development was silence, see Figure 3.

**Figure 4:**
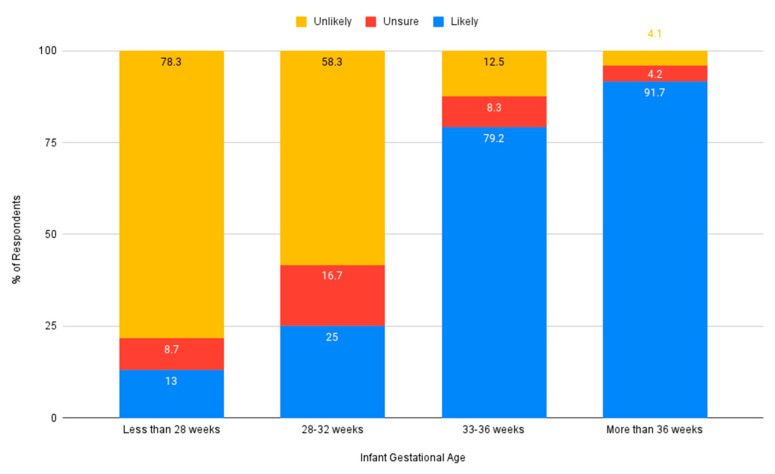
Likeliness to advocate for an auditory intervention based on gestational age.

**Figure 5:**
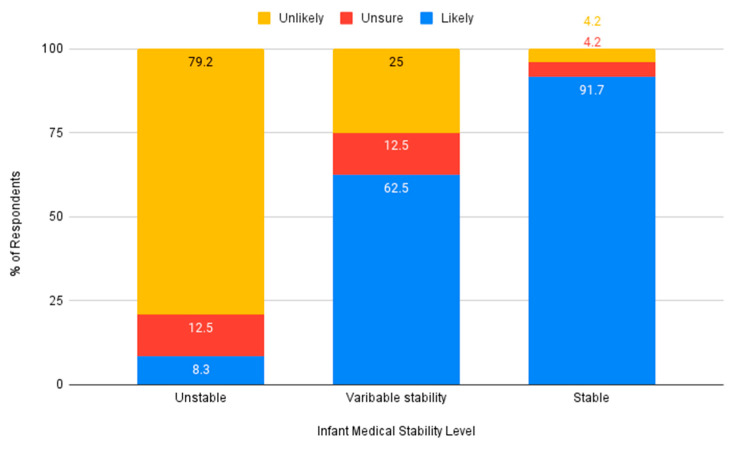
Likeliness to advocate for an auditory intervention based on medical stability.

Nurses were asked if they believed an auditory intervention was needed in the NICU. The majority of respondents indicated they believed auditory intervention for premature infants would be beneficial (*n* = 42, 91.3%). Nurses indicated that they would be more likely to advocate for auditory intervention for moderately premature or term infants, compared to very premature infants (see Figure 4). Similarly, respondents indicated they would be more likely to advocate for an auditory intervention for infants who have variable stability or who are stable, and less likely to do so for infants who are medically unstable (see Figure 5).

Nurses are the primary professional caregivers who spend the most amount of time at the bedside of premature infants, so we were interested to know if nurses would be willing to sing to infants as a method to meet early auditory development needs. Over two-thirds of nurses (*n* = 15, 79.5%) reported that they would be willing to sing to premature infants.

### 3.4. Exploration of Nurse Age and Experience on Voice Behaviors and Beliefs

**Figure 6:**
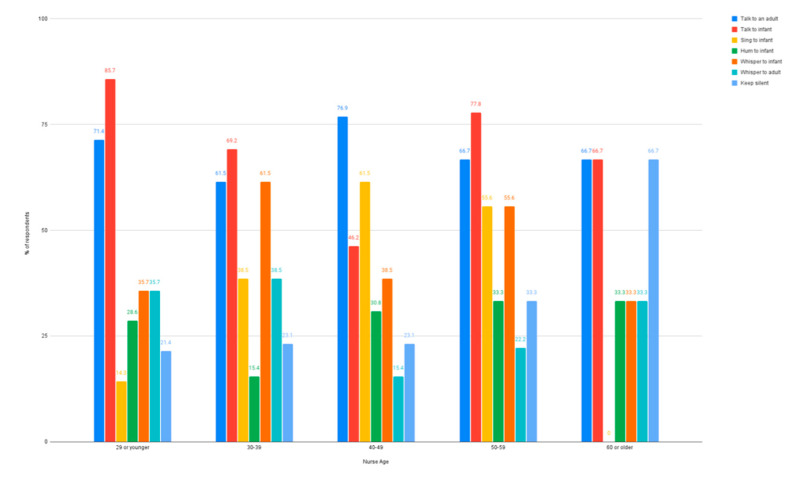
Types of voice use separated by the age of the nurse.

The influence of age on how nurses use their voice in the NICU was examined. Among nurses who were 40–49 years old, less than half (*n* = 6, 46.2%) indicated they used their voice to talk to an infant, while more than half of respondents from all other age groups reported using their voices to talk to an infant. Those 40–49 years old did, however, indicate they sang to infants when providing bedside care at a higher percentage (*n* = 8, 61.5%) than any other age range (14.3–55.6%). Humming to an infant was the lowest reported type of voice use, although it was present across every age group (15.4–33.3%). Whispering to an infant was highest for nurses in their 30 s (*n* = 8, 61.5%) and 50 s (*n =* 5, 55.6%). Using the voice to whisper to other adults was low across all age groups (15.4–38.5%) and keeping silent was reported as more common in nurses who were 60 or older (*n* = 2, 66.6%). Figure 6 illustrates patterns across each nurses’ age group reporting typical voice use while caring for infants in the NICU.

Similarly, the influence of years of experience working in the NICU on voice use was examined. Nurse respondents with 20 years or more experience in the NICU reported the most voice use directly with infants in a variety of ways such as talking (*n* = 13, 86.7%), singing (*n* = 8, 53.3%), humming (*n* = 8, 53.3%), or whispering (*n* = 8, 53.3%). While nurses with only 5–10 years of experience similarly used their voice in a variety of ways to talk to infants (*n* = 8, 67%), sing to infants (*n* = 4, 50%), and whisper to infants (*n* = 4, 50%), nurses with 5–10 years of experience were much less likely to hum to infants (*n* = 1, 12.5%). At over 75%, talking to the infant was reported as the most common type of voice use across all levels of experience. Over 50% of nurses in all experience levels reported talking to adults; among nurses with 5–15 years of experience, whispering to an adult (*n* = 9, 56.3%) or keeping silent (*n* = 7, 43.8%) were the most common.

**Figure 7:**
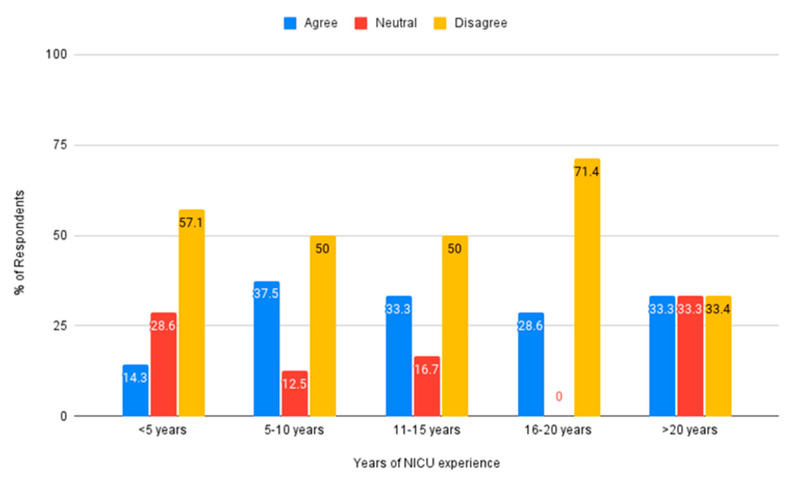
Belief that the auditory environment is sufficient to meet auditory needs by nurse years of experience in the NICU.

The impact of years of experience in the NICU was also used to explore nurse beliefs about the auditory environment. Across all levels of experience, the majority of nurses reported believing the NICU environment is not sufficient to meet the auditory needs of premature infants, see Figure 7.

## 4. Discussion

The purpose of the current study was to examine how nurses self-report using their voice while caring for premature infants in the NICU and what they believe about NICU infants’ early auditory development/needs. To our knowledge, this is the first study to directly examine these factors. Understanding NICU nurse beliefs and behaviors is an important preliminary step for improving current care standards, as well as for developing and evaluating evidence-based interventions that target infants’ auditory and language development in the NICU.

Voice sounds and language input are salient features of the intrauterine environment that are essential for organization of the infants’ auditory system [6,20] and critical for supporting optimal language development [19,29,30]. We found that NICU nurses report using their voices in a variety of ways—including speaking, singing, or whispering—as they provide care to premature infants. The primary form of voice use reported by nurses in this study was speaking, both to infants and other adults at the bedside. What nurses may have been doing at the bedside (i.e., changing a diaper, feeding, taking vital signs, or giving medication) influenced their voice behaviors. During more routine caregiving tasks like feeding or changing a diaper, nurses were more likely to report using their voice; in comparison, during tasks like giving medication or taking vital signs, which are more medical in nature, nurses reported using their voice less often. Additionally, nurses reported that they change the sound of their voice when interacting with premature infants compared to how they speak to adults.

A key finding of this study was the reported increase in nurses voice use with premature infants as their medical stability improved and they became closer to term gestational age. Infant behavioral cues also influenced nurses’ voice use. Given the historical practice of limiting sensory stimulation for very premature infants in the NICU, this was not a surprising result. As premature infants approach term gestation they may be more medically stable, able to integrate increased sensory stimulation, and display behaviors that are more akin to those of a full-term infant. Providing care to infants who more readily provide cues such as eye contact or cooing more easily elicits adult responding and likely motivates nurses to naturally increase their vocal interactions [31].

The propensity of nurses to use their voices more with older and more stable infants suggests that NICU nurses may be providing an auditory experience that supports language development for those infants [23]. However, with a critical period of auditory development occurring during the third trimester, before infants reach term, protecting premature infants from voice sounds while in the NICU could negatively impact both auditory and language development [3,4]. Recent research using early application of positive sensory experiences for premature infants shows promise for improving language outcomes [32]. In the current survey, nurses reported a tendency to whisper or be in silence for infants less than 28 weeks gestational age. Nurses’ bias to whisper to very premature infants may be an intentional strategy for reducing auditory experiences perceived as being too stimulating for the infant to handle. While whispering may not be a harmful auditory experience, whispering may lack important voice characteristics of pitch and prosody that carry the preliminary characteristics necessary for language development during the critical period of the third trimester auditory experience [6,19,20]. Little is known about how whispering may impact premature infant auditory perception and language development, although one study found newborn infants did not show preference for maternal whispered voices when compared to maternal low-pass filtered voices meant to simulate those found in the uterine environment [33]. Other types of voice use (singing and humming) were reportedly used less often by nurses and followed the same trend as talking, increasing as the gestational age increased and medical stability improved. The innately musical characteristics of language as it is filtered into the intrauterine environment and in infant-directed speech may provide a strong rationale for how musical voice sounds, such as singing, could be a pathway for enhancing the early auditory experience of premature infants [34].

In this study, approximately half of the nurses surveyed did not believe the environment of the NICU was sufficient to support auditory development of premature infants, although they did believe that infants had sufficient exposure to voice sounds. Surveyed nurses identified background noise as both a barrier to voice exposure and as having a negative impact on auditory development. In contrast, silence was not identified as a barrier to voice exposure but was viewed as having a negative impact on auditory development. This finding demonstrates a possible gap in understanding regarding the role of voice exposure in early auditory development. Our results indicate the need for specific education on the relationship between the environment, voice use, and early auditory development is warranted, particularly since the acoustic nature of the NICU environment is a constant topic of concern for its link to long-term developmental difficulties in premature infants [13,17,18].

Decibel levels within the NICU have been targeted for change within developmental care practices for decades, and in this study, background noise was believed to prevent premature infants from hearing voice sounds. NICU designs have shifted to private room layouts in an attempt to reduce exposure to harmful sounds and sustained noise, among other things, but silence has also come to the forefront for its potentially negative impact on development [11]. Prolonged silence in the NICU environment has been associated with infants’ lower language skills [35,36]. Access to meaningful language is difficult in both open bay and private room NICUs; however, private rooms are quieter, which is believed to allow for potential access to more language [35]. NICU design is an important factor that must be considered for its impact on the quality of the auditory environment.

NICU design did influence nurses’ perceptions of how the auditory environment meets the needs of premature infants. Nurses working in open bay and combination units were more likely to believe the overall environment was not sufficient for auditory development due to constant background noise, whereas nurses in private rooms were split on their beliefs of the auditory environment being sufficient or not. Nurses in this study who indicated working in a private room NICU had a more favorable view of parents providing sufficient access to voice sounds, but those from open bay NICUs indicated parents did not provide sufficient voice sound exposure. Those working in combination NICUs were more equally distributed in their beliefs of parent voice use. Parent presence and interaction with the infant is associated with improved developmental outcomes; however, social emotional and sociodemographic circumstances may limit parents’ ability to be present and interactive with their premature infant [37]. Language exposure has been shown to increase only when parents are actively holding their infant compared to being present at the bedside without holding [38]. Additionally, it has been demonstrated that premature infants receive more language exposure during staff care than when parents are providing care, which may be an indicator of the impact emotional stress plays on parent–infant interactions in the NICU [39]. While parents’ presence tends to be reported as higher in private room NICUs, the presence of the parent alone does not increase exposure to language [21]. Nurse respondents’ beliefs about voice use in different NICU settings align with reported parent presence in the previous literature but indicate a need for future investigation of interventions that increase parent voice use with their child in the NICU.

Nurses identified speaking and singing (both voice sounds), as well as recorded womb sounds and recorded instrumental music (both non-voice sounds), as having the greatest impact on auditory development. In contrast, conversation (voice sound) and silence (non-voice sound) were identified as having the greatest negative impact on auditory development in premature infants in the NICU. Silence is a barrier to language development [12,40], given the substantial research linking amount/quality of language input to child language outcomes in term infants, and given emerging research demonstrating the same relationship between exposure to adult conversation in the NICU and later language development [41]. While auditory intervention was strongly supported by nurses in this survey, they were more likely to advocate for such intervention for moderately premature or term infants who were medically stable. With increasing evidence to support the connection between voice use and language development in premature infants, the reported trend in surveyed nurses to wait until closer to term demonstrates a possible missed opportunity to provide essential auditory input during a critical period for premature infants (1,2). This also is an important potential barrier to consider for future intervention efforts.

Developmental care, a broad term originally used to describe interventions aimed at minimizing stressors related to the NICU [42], is evolving with the growing understanding of the need for intervention to enhance the NICU environment [43]. NICU nursing is a specialized subset of the nursing profession that is primarily learned on the job after more general nursing education [44]. For this reason, the age and years of NICU nursing experience may potentially play a role in nurses’ beliefs about infant auditory needs and voice behaviors during infant interactions. Evidence suggests that years of experience working in the NICU may be a predictor of a nurse’s use of developmental care practices at the bedside and having more years of experience tends to increase the practice of developmental care [45]. This trend was reflected in the current study, where nurses with 20 or more years of experience most often reported using their voice in a variety of ways with infants in the NICU. Experience may be an indicator of a shift in beliefs about what kind of auditory experiences premature infants need and/or a change in understanding about the kinds of adult–infant interactions that can be supportive for premature infants. Interestingly, nurses with less experience believed that the NICU environment was not optimal for premature infant auditory development, but this belief was not reflected in their self-reported voice use behaviors. This discrepancy could be driven by less experience/lower confidence in deploying different voice use behaviors with premature infants. Further investigation into the voice use decision making of experienced nurses is warranted to understand the process involved in the influence of changing beliefs on bedside practices. Nurse age had a less pronounced influence on beliefs and behaviors, which may further indicate that years of experience in the NICU may be a primary driver of nursing practices.

Overall, NICU nurses agreed that an intervention to target the auditory development of premature infants is needed. They also indicated that they would refer premature infants for an auditory intervention, although this was primarily only for older gestational age infants. The same infant characteristics of age and medical stability that influenced nurse voice use also influenced their perception of whether infants would be appropriate for intervention. This is a barrier that will be important to address because younger premature infants, who are in the critical period of foundational auditory development, would potentially miss out on beneficial auditory intervention.

Nurses in this study indicated that even though they may not currently use their voice to sing or hum to premature infants, they would be willing to sing to meet the auditory needs of their patients. Based on reviews of previous research and theoretical modeling, characteristics of an auditory intervention that should be considered (throughout the development and evaluation of evidence-based practices) should include voice sounds that mimic certain features of the intrauterine environment, such as pitch and language prosody, through singing and humming [34]. Engaging nurses in education regarding the trajectory of early auditory development, safety of certain voice sounds, and the possible impact of voice on auditory development may allow nurses to feel more comfortable using their own voice. Additional studies are needed to develop evidence-based intervention components that target early auditory and language development as well as intervention approaches that target enhancing nurse knowledge about premature auditory development and voice behaviors. For example, historical trends to reduce noise in the NICU and lack of knowledge about the kinds of auditory/language interactions that support premature infant development may inadvertently produce a sound-deprived NICU environment that dampens developmental outcomes.

The current study represents a foundational first step in examining NICU nurses’ voice behaviors and beliefs about auditory development for premature infants. Understanding how NICU nurses report calibrating their voice use behaviors, as a function of the infants’ medical and behavioral state, gestational age, and other factors, is a critical step for basic research (to examine the kinds of sound experiences that support/impede premature auditory development in the NICU) and translational studies (to develop and evaluate interventions, both at the NICU level and patient level). It will be important to replicate the current findings in a larger sample of NICU nurses, as well as including other health care providers, which is highly feasible for this survey design. A panel of NICU nurses offered their expertise on the survey questionnaire prior to recruitment. We ran a pilot of the survey questionnaire that offered slight revisions for clarity followed by a second level of recruitment. Outcomes from both survey recruitment periods were well “matched” so we could combine the results. The levels of review and revision to support the construction of the questionnaire and the content and experience expertise of the researchers are additional strengths of this study.

The current study includes limitations that may impact the interpretation of the results. Due to the use of social media to recruit participants through a self-selected anonymous link, a response rate could not be calculated. Based on the reported membership of the National Association of Neonatal Nurses (NANN), 73 responses may represent only approximately 1% of neonatal nurses. Two recent studies targeting practicing neonatal nurses reported response rates between 2% and 4% [46,47]. Response rates across all distribution types are declining due to increases in refusals, and nurses are known to be difficult to survey due to survey fatigue and hospital structures that prevent access to online surveys [47,48]. Opt-in surveys, like the one used in this study, may have a high incidence of topic bias as participants with a specific interest in the topic are more likely to complete the survey [49]. Additionally, nurses in this study were asked to self-report how they use their voice while providing care in the NICU, which may not be an accurate representation of their actual behavior. Finally, given the modest sample size, it is important to interpret the generalizability of results with caution. It will be important to replicate current results in a larger sample of nurses that also includes non-U.S. participants.

The results of this study provide an important preliminary understanding of what NICU nurses currently believe about premature infant auditory development and how they report using their voices while in the NICU. There may be specific ways that the voice can be used to maximize exposure to speech sounds that are salient in the intrauterine environment yet absent from the extrauterine environment of the NICU. The reported voice behaviors of NICU nurses suggest the need for further investigation of the relationship between infant responses and different auditory experiences at varying gestational ages and levels of medical stability. This would inform evidence-based practice behaviors of auditory experiences provided by professional and family caregivers in the NICU. Nurses voice use and perceptions of the auditory needs of premature infants also indicate that additional nurse education on the possible benefits of voice use with younger premature infants, as well as using a variety of voice sounds, would be necessary to broaden the current voice behaviors of NICU nurses.

Additional study is needed to compare the self-reported data collected in this study with observational data of nurse voice use behaviors at the bedside of premature infants. Habituation to sounds in the environment occurs as a result of working for long periods in the NICU and may impact the perception of voice use compared to actual voice use. For example, nurses may be talking more at the bedside of younger infants than they perceive based on communication needs or workflow. Naturalistic observations of nurse voice behaviors will help highlight the impact that nurses can have on early auditory development by bringing awareness to current voice use patterns, factors influencing voice use decisions, and opportunities for change. Continuing to gauge nurses’ self-reported responses with direct observational data will help to strengthen the understanding of voice use and beliefs and provide a foundation for possible intervention pathways.

## 5. Conclusions

This study is the first to examine NICU nurses’ beliefs and perceptions of their voice use with premature infants in the NICU. Results found that NICU nurses report using their voices mostly for speaking at the bedside of premature infants who are close to term gestational age. The surveyed nurses do not believe the auditory environment of the NICU is sufficient to support premature infant auditory development, but a gap in knowledge regarding the importance of early voice exposure may be a barrier to nurses using their voice with younger infants who are in a critical period of auditory development. This study is a preliminary exploration into the need of a targeted auditory intervention from the perspective of NICU nurses and provides an emerging understanding of how nurses may benefit from additional education and training on a targeted intervention that supplements auditory experiences for language development. Future inquiry is needed that involves observations or recordings of NICU nurse voice use for further analysis in order to confirm whether nurse behaviors are aligned with the self-reported data. Interview studies that seek to discover a deeper insight into the nuances of NICU nurse decision making will also be important to the aim of mitigating the long-term language outcomes of premature infants.

## Figures and Tables

**Table 1:** Description of respondent demographics.

Question	Sum Responses (%)
Number of years as an RN (*n* = 57)	
Less than 5	18 (31.6)
5–10	9 (15.8)
11–15	4 (7.0)
16–20	5 (8.8)
More than 20	21 (36.8)
Number of years as an RN in the NICU (*n* = 57)	
Less than 5	20 (35.1)
5–10	8 (14.0)
11–15	8 (14.0)
16–20	8 (14.0)
More than 20	13 (22.8)
Age (*n* = 63)	
24 or younger	5 (7.9)
25–29	12 (19.0)
30–34	10 (15.9)
35–39	5 (7.9)
40–44	12 (19.0)
45–49	5 (7.9)
50–54	4 (6.3)
55–59	7 (3.2)
60–64	2 (3.2)
65 or older	1 (1.6)
Highest nursing degree (*n* = 58)	
Associates	6 (10.3)
Bachelors	42 (72.4)
Masters	7 (12.1)
Doctorate	3 (5.2)
Gender (*n* = 63)	
Female	60 (95.2)
Male	3 (4.8)
Race (*n* = 63)	
American Indian/Alaskan	2 (3.2)
Asian	3 (4.8)
White	56 (88.9)
Two or more races	2 (3.2)
Ethnicity (*n* = 72)	
Hispanic/Latino	7 (11.3)
Non-Hispanic/Latino	55 (88.7)
U.S. Geographical Region (*n* = 55)	
West	6 (10.9)
Midwest	27 (49.1)
Southwest	13 (23.6)
Southeast	5 (9.1)
Northeast	4 (7.3)
NICU Location (*n* = 54)	
Urban	36 (66.7)
Suburban	15 (27.8)
Rural	3 (5.6)

**Table 2:** Description of reported unit characteristics.

Question	Sum Response (%)
Designation level of the NICU where you are employed (*n* = 53)	
Level II	6 (11.3)
Level III	21 (39.6)
Level IV	26 (49.1)
Design of the NICU where you are employed (*n* = 54)	
Open Bay	12 (22.2)
Private Room	22 (40.7)
Combination	20 (37.0)
Developmental Services Present in the NICU where you are employed (*n* = 55)	
Social Work	49 (89.1)
Physical Therapy	46 (83.6)
Occupational Therapy	41 (74.5)
Developmental Care Team	37 (67.3)
Speech Therapy	37 (67.3)
Child Life	31 (56.4)
Music Therapy	25 (45.5)
NIDCAP Certified	12 (21.8)
Psychology	8 (14.5)

## Data Availability

Data presented in this study are available upon request from the corresponding author. The data are not publicly available due to the participant consent form not indicating publication of the raw dataset.

## Supplementary Materials

The following are available online at https://www.mdpi.com/article/10.3390/ijerph18168471/s1, Table S1: Questionnaire revisions, Table S2: Final questionnaire.
