# Supplementary material for: A Survey of Neonatal Nurses Perspectives on Voice Use and Auditory Needs with Premature Infants in the NICU
Source: Int J Environ Res Public Health. 2021 Aug 11;18(16):8471. doi: 10.3390/ijerph18168471 (PMC8393431; doi:10.3390/ijerph18168471)
Supplement: Supplementary file 1 [file ijerph-18-08471-s001.zip › ijerph-1276336-supplementary/S1_Questionnaire Revisions.pdf]

Question	Version 1	Revised Version 2
For each type of caregiver below, indicate how **similar or different their beliefs about auditory needs are to your own.**	Sliding scale response (0 = very different from me; 100 = very similar to me)	Likert response (very similar to me, somewhat similar to me, unsure, somewhat different than me, very different than me)
Indicate to what degree you feel confident about the sound of your singing voice	Sliding scale (0 = not confident; 100 = very confident	**Revised Question:** I feel confident in the sound of my singing voice.<br><br>Likert response (strongly agree, agree, neither agree or disagree, disagree, strongly disagree)<br><br>**Additional Question:** Overall how would you describe parent presence in your NICU? (frequently present, sometimes present, rarely present)
