# Supplementary material for: A Survey of Neonatal Nurses Perspectives on Voice Use and Auditory Needs with Premature Infants in the NICU
Source: Int J Environ Res Public Health. 2021 Aug 11;18(16):8471. doi: 10.3390/ijerph18168471 (PMC8393431; doi:10.3390/ijerph18168471)
Supplement: Supplementary file 1 [file ijerph-18-08471-s001.zip › ijerph-1276336-supplementary/S2_Final Questionnaire.pdf]

Category	Question	Response Options
Voice Use		
	During a typical day while providing bedside care to infants how do you **most often** use your voice (select all that apply)	Talk to another adult nearby; talk directly to the infant, sing to the infant, hum to the infant, whisper to the infant, whisper to another adult nearby, keep silent, I don't provide care.
	How often does the age of the infant you are caring for influence your voice use?	Always, most of the time, 1/2 of the time, sometimes, never
	How often does the medical stability of the infant influence your voice use?	Always, most of the time, 1/2 of the time, sometimes, never
	How often do the individual cues of the infant influence your voice use?	Always, most of the time, 1/2 of the time, sometimes, never
	When I am providing bedside care for extremely preterm infants (less than 28 weeks) I typically do the following with my voice:	Talk to another adult nearby; talk directly to the infant, sing to the infant, hum to the infant, whisper to the infant, whisper to another adult nearby, keep silent, I don't provide care.
	When I am providing bedside care for very preterm infants (28-32 weeks) I typically do the following with my voice:	Talk to another adult nearby; talk directly to the infant, sing to the infant, hum to the infant, whisper to the infant, whisper to another adult nearby, keep silent, I don't provide care.
	When I am providing bedside care for moderate to late preterm infants (33-36 weeks) I typically do the following with my voice:	Talk to another adult nearby; talk directly to the infant, sing to the infant, hum to the infant, whisper to the infant, whisper to another adult nearby, keep silent, I don't provide care.
	When I am providing bedside care for term to post term infants (37weeks and older) I typically do the following with my voice:	Talk to another adult nearby; talk directly to the infant, sing to the infant, hum to the infant, whisper to the infant, whisper to another adult nearby, keep silent, I don't provide care.
	Indicate how likely you are to use your voice with a medically stable infant (Room air or minimal oxygen support, no restrictions to being held by caregivers or family, no variation in vital signs with stimulation or position changes)	Extremely likely; somewhat likely neither likely or unlikely, somewhat unlikely; extremely unlikely

	Indicate how likely you are to use your voice with an infant who has variable stability (patient may be on ventilator or oxygen support but only minimal changes in vital signs with position changes or stimulation, consideration and caution taken prior to moving patient out of bed for holding but patient is able to be held)	Extremely likely; somewhat likely neither likely or unlikely, somewhat unlikely; extremely unlikely
	Indicate how likely you are to use your voice with a medically unstable infant (intubated/ventilator support or ECMO or requiring high level of supplemental oxygen, significant changes in vital signs noted with position changes or stimulation, patient on continuous medication infusions (e.g., vasopressors or sedation), patient unable to be held)	Extremely likely; somewhat likely neither likely or unlikely, somewhat unlikely; extremely unlikely
	I would use my voice with an infant who is restless	strongly agree, somewhat agree, neither agree/disagree, somewhat disagree, strongly disagree
	I would use my voice with an infant who is alert	strongly agree, somewhat agree, neither agree/disagree, somewhat disagree, strongly disagree
	I would use my voice with an infant who is crying	strongly agree, somewhat agree, neither agree/disagree, somewhat disagree, strongly disagree
	During a typical day caring for preterm infants, I use my voice when I am (select all that apply):	Changing the diaper, assessing vital signs, feeding, giving medication, performing a procedure, other, I don't use my voice during infant care (provide a reason)
	I change the way my voice sounds when interacting with an infant I am caring for compared to when I interact with other adults in the NICU	Yes/No
Beliefs/knowledge about auditory needs		
	I believe the overall auditory development needs of infants in the NICU where I work are sufficiently met	strongly agree, somewhat agree, neither agree/disagree, somewhat disagree, strongly disagree

Rate the impact you believe each voice sound has on auditory development: adult to adult conversation, live parent voice, recorded parent voice, live voices other than parents, recorded voices other than parents, live singing (no instruments), recorded singing (no instruments)	Positive impact, neutral impact, negative impact
Rate how valuable to auditory development the following types of non-voice sounds are in the NICU: Silence, live instrumental music (no voice); recorded instrumental music (no voice); music from mobiles; recorded womb sounds, recorded white noise	Positive impact, neutral impact, negative impact
I believe infants in the NICU are exposed to a sufficient amount of voice sounds to meet their auditory needs	strongly agree, somewhat agree, neither agree/disagree, somewhat disagree, strongly disagree
In the NICU where I work, I believe environmental background noise prevents infants from hearing voice sounds to meet auditory needs	strongly agree, somewhat agree, neither agree/disagree, somewhat disagree, strongly disagree
In the NICU where I work, I believe it is too quiet which prevents infants from hearing voice sounds to meet auditory needs	strongly agree, somewhat agree, neither agree/disagree, somewhat disagree, strongly disagree
In the NICU where I work, I believe parents provide sufficient exposure to voice sounds to meet the auditory needs of their infant	strongly agree, somewhat agree, neither agree/disagree, somewhat disagree, strongly disagree
I believe an intervention is needed to improve the auditory development of infants who are	strongly agree, somewhat agree, neither agree/disagree, somewhat disagree, strongly disagree
I would advocate for infants in the NICU to receive a singing intervention from a music therapist to meet auditory development needs at these ages	Less than 28 weeks; 28-32weeks; 33-36weeks; 34-36weeks; 37weeks or older (select multiple)
I would consider singing to infants in the NICU to meet their auditory development needs	strongly agree, somewhat agree, neither agree/disagree, somewhat disagree, strongly disagree
For each type of caregiver below, indicate how similar or different their beliefs are about auditory needs to your own: Other nurses, physicians, therapists, parents	Very similar, similar, unsure, different, very different

	I would like to share these additional thoughts about voice use or auditory needs	comment:
Demographics/Background		
	Age	20-24; 25-29; 30-34; 35-39; 40-44; 45-49; 50-54; 55-59, 60-64; 65+
	Gender	Female, male, trans male, trans female, non-binary, prefer not to say, not listed
	Ethnicity	Hispanic/Latino; non-Hispanic/Latino
	Race	American Indian or Alaskan Native; Asian; Native Hawaiian or other Pacific Islander; Black or African American; White; Two or more races
	Highest degree	Associate, Bachelors, Masters, Doctoral
	Specialized nursing certification	APRN, CCRN, CPN, NNP…other
	Total years of nursing practice (any unit)	<5; 5-10; 11-15; 16-20, more than 20
	Total years of nursing practice in the NICU	<5; 5-10; 11-15; 16-20, more than 20
	Did you learn about auditory development during your nursing education	Yes/No
	Have you recently (in the past 5 years) learned about infant auditory development in a seminar, presentation or continuing education format?	Yes/No
	I feel confident in my current knowledge of infant auditory development	strongly agree, somewhat agree, neither agree/disagree, somewhat disagree, strongly disagree
	In your personal life do you, or have you in the past, had more than occasional experience with infants (i.e., as a nanny, caregiver, parent)	Yes/No
	I have observed a music therapist providing services in the NICU	Yes/No
	I have read research about music therapy in the NICU	Yes/No
	I have attended a seminar or conference presentation about music therapy in the NICU	Yes/No
	I have read about music therapy and the NICU in the news.	Yes/No

I have watched a video about music therapy in the NICU	Yes/No
In the past, I have taken music lessons or participated in a music group such as a choir or band	Yes/No
I am currently taking music lessons or participating in a music group such as a choir or a band	Yes/No
I feel confident in the sound of my own voice	strongly agree, somewhat agree, neither agree/disagree, somewhat disagree, strongly disagree
The municipal location of the NICU where I currently work is:	Urban, suburban, rural
Using the map provided: the NICU where I currently work is in what region of the United States:	West, Midwest, Southwest, Southeast, Northeast, International country________
The level designation of the NICU where I currently work is	Level IV, Level III, Level II
The unit design of the NICU where I currently work is:	Open bay, private room, combined, other:
Developmental services present in the NICU where I currently work are:	Newborn Individualized Developmental Care and Assessment Program (NIDCAP)-Certified; developmental care team or committee; developmental physician, developmental rounds, other:
In addition to nurses, staff present in the NICU where I currently work include:	Occupational therapists; Physical therapists, speech therapists, music therapists, developmental specialist, child life specialist, psychologist, social worker other:
In the unit where you work how would you describe parent presence?	Frequently present (most days of the week or long duration of time), sometimes present (a few days a week or variable duration of time, rarely present (not weekly or very short duration of time)
